# Longitudinal and Vertical Variations of Dissolved Labile Phosphoric Monoesters and Diesters in the Subtropical North Pacific

**DOI:** 10.3389/fmicb.2020.570081

**Published:** 2021-01-20

**Authors:** Tamaha Yamaguchi, Mitsuhide Sato, Fuminori Hashihama, Haruka Kato, Takanori Sugiyama, Hiroshi Ogawa, Kazutaka Takahashi, Ken Furuya

**Affiliations:** ^1^Fisheries Resources Institute, Japan Fisheries Research and Education Agency, Yokohama, Japan; ^2^Department of Aquatic Bioscience, Graduate School of Agricultural and Life Sciences, The University of Tokyo, Tokyo, Japan; ^3^Graduate School of Fisheries and Environmental Sciences, Nagasaki University, Nagasaki, Japan; ^4^Department of Ocean Sciences, Tokyo University of Marine Science and Technology, Tokyo, Japan; ^5^Atmosphere and Ocean Research Institute, The University of Tokyo, Chiba, Japan; ^6^Graduate School of Science and Engineering, Soka University, Tokyo, Japan

**Keywords:** subtropical ocean, dissolved organic phosphorus, monoesters, diesters, alkaline phosphatase activity

## Abstract

The labile fraction of dissolved organic phosphorus (DOP) – predominantly consisting of phosphoric esters – is an important microbial P source in the subtropical oligotrophic ocean. However, unlike phosphate, knowledge for labile DOP is still limited due to the scarcity of broad and intensive observations. In this study, we examined the concentrations and size-fractionated hydrolysis rates of labile phosphoric monoesters and diesters along a >10,000 km longitudinal transect in the North Pacific (23°N; upper 200-m layer). Depth-integrated monoesters decreased westward with a maximum difference of fivefold. Vertical profiles of monoesters in the eastern and western basins showed decreasing and increasing trends with depth, respectively. The monoester-depleted shallow layer of the western basin was associated with phosphate depletion and monoesterase activity was predominant in the large size fraction (>0.8 μm), suggesting that monoesters are significant P sources particularly for large microbes. In contrast, diester concentrations were generally lower than monoester concentrations and showed no obvious horizontal or vertical variation in the study area. Despite the unclear distribution pattern of diesters, diesterase activity in the particulate fraction (>0.2 μm) increased in the phosphate-depleted shallow layer of the western basin, suggesting that the targeted diesters in the assay were also important microbial P sources. Diesterase activities in the dissolved fraction (<0.2 μm) were not correlated with ambient phosphate concentrations; however, cell-free diesterase likely played a key role in P cycling, as dissolved diesterase activities were substantially higher than those in the particulate fraction. The horizontal and vertical variability of labile monoesters in the subtropical North Pacific were therefore predominantly regulated by P stress in particularly large microbes, whereas the distributions of labile diesters and diesterase activities were generally independent of microbial P stress, indicating a more complex regulation of diesters to that of monoesters.

## Introduction

Dissolved organic phosphorus (DOP) comprises a significant portion of dissolved phosphorus (P) in the surface layer of the subtropical ocean (Paytan and McLaughlin, 2007; Karl and Björkman, 2015). Since phosphate is scarce at nanomolar levels therein (Wu et al., 2000; Hashihama et al., 2009), DOP plays an important role as an alternative P source for microbes. DOP is predominantly mediated by microbes – mainly phytoplankton – in the surface layer through processes such as cell excretion, exudation, and release from damaged cells (Kuenzler, 1970; Orrett and Karl, 1987; Young and Ingall, 2010). Thus, DOP comprises various organic P compounds involved in living organisms, including phospholipids, nucleic acids, and sugar phosphates (Karl and Björkman, 2015). Based on their chemical structures, DOP compounds are typically grouped into phosphoric esters and phosphonates and phosphoric esters contribute the highest proportion (80–85%) to the total DOP pool in various oceanic regions (Young and Ingall, 2010).

In general, microbes express alkaline phosphatases and hydrolyze phosphoric esters under P limitation to meet their cellular P demands (Kuenzler and Perras, 1965; Yentsch et al., 1972). Compiled field studies have also demonstrated a distinct elevation of monoesterase activity (MEA) under low phosphate conditions in both the Atlantic and Pacific Oceans (Mahaffey et al., 2014 and references therein). Based on these findings, the labile fraction of phosphoric monoesters has been considered as an important P source for microbes, and studies have increasingly reported the distribution of monoesters in the oligotrophic ocean (Moutin et al., 2008; Duhamel et al., 2010; Suzumura et al., 2012; Hashihama et al., 2013, 2019; Sato et al., 2013; Vidal et al., 2018; Yamaguchi et al., 2019). Although the examined oceanic regions and depths are still limited, monoester concentrations are mostly below or around the detection limit in the upper 100-m layer and contribute a small proportion of the total DOP pool.

However, the application of highly sensitive colorimetry identified significant regional variability in the vertical profiles of nanomolar monoesters. Studies have revealed increasing and decreasing trends with depth in the western North Pacific (Hashihama et al., 2013) and the central North Pacific (Yamaguchi et al., 2019), respectively. Furthermore, from a horizontal viewpoint, lower monoester concentrations in conjunction with lower phosphate concentrations were observed in the subtropical North Pacific (Hashihama et al., 2013, 2019; Yamaguchi et al., 2019), whereas higher monoester concentrations in conjunction with lower phosphate concentrations were observed in the subtropical and temperate North Atlantic (Vidal et al., 2018). These results reflect the complex regulation of labile monoesters in the global oceans. It is therefore necessary to further investigate the distribution of labile monoesters in various environmental circumstances.

Here diesters as well as monoesters are important components of the bioavailable fraction of phosphoric esters. They are an essential component of cell membranes and nucleic acids in living organisms and likely play a significant role in the biologically mediated DOP. The scarcity of diesters (Vidal et al., 2018; Yamaguchi et al., 2019) and the stimulation of diesterase activity (DEA) under phosphate-depleted conditions (Sato et al., 2013; Yamaguchi et al., 2019) suggest that diesters are also important microbial P sources. However, most studies have focused on the distributions of monoesters, while the dynamics of diesters in the subtropical ocean are still poorly understood.

Compared to studies on the distribution of labile phosphoric ester concentrations, alkaline phosphatase activities have been investigated as an indicator of microbial P stress since the early times (Perry, 1972; Hoppe, 2003; Karl, 2014). As alkaline phosphatases are expressed by all living organisms (Karl, 2014), it is ecologically important to identify the specific organisms particularly utilizing phosphoric esters. The size fractionation of samples is an effective approach to reveal this point; however, studies reporting multiple separations of particulate-associated MEA and DEA are still limited in the open ocean (Vidal et al., 2003; Hoch and Bronk, 2007; Duhamel et al., 2010). Furthermore, dissolved enzyme activities, which are discriminated from the particulate enzyme activities, provide additional information on the current or recent P-specific physiological states of microbes due to the relatively long lifetime of cell-free dissolved alkaline phosphatases (Li et al., 1998; Baltar et al., 2013). To date, the reported dissolved phosphatase activities vary considerably from 6 to 100% of the bulk phosphatase activity in the North Pacific (Hoch and Bronk, 2007; Duhamel et al., 2010, 2011; Sato et al., 2013); however, the controlling factors of this high fluctuation remain unclear.

In this study, we examined the concentrations and size-fractionated hydrolysis rates of monoesters and diesters in the subtropical North Pacific to elucidate the underlying mechanisms influencing the dynamics of labile phosphoric ester. The study area covered a distinct longitudinal gradient of ambient phosphate concentrations (Hashihama et al., 2009; Martiny et al., 2019); this enabled us to verify the ubiquity of previously reported characteristics of labile phosphoric esters under various P-specific conditions throughout the subtropical ocean.

## Materials and Methods

### Field Observations and Environmental Parameters

**FIGURE 1:**
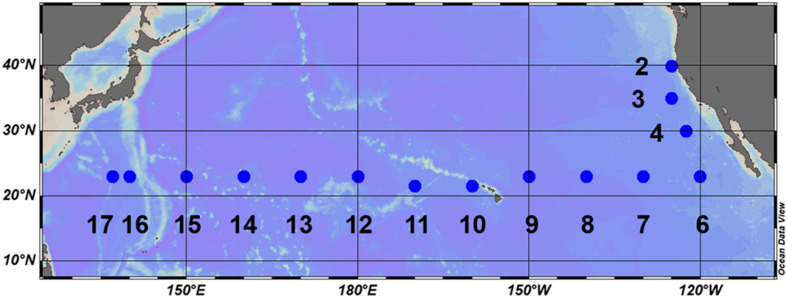
Location of stations during the KH-17-4 cruise (August–October 2017).

Field observations were conducted during the KH-17-4 cruise (August 12 to October 5, 2017) on-board the R/V Hakuho-Maru. Three stations were located off the California coast and 12 stations were located in the subtropical North Pacific along a 23°N transect (Figure 1). The stations along the transect were separated at intervals of approximately 10° longitude. Vertical profiles of light intensity were measured using a Hyper Profiler (Satlantic LP, Halifax, NS, Canada) and depths corresponding to 25, 10, 1, and 0.1% of the surface light intensity were determined. Depth profiles of temperature were measured using an SBE 911 plus conductivity-temperature-depth (CTD) system (Sea-Bird Electronics, Inc., Bellevue, WA, United States). Water samples were collected using an acid-cleaned bucket or 12-L Niskin-X bottles fitted with a rosette system attached to the CTD.

### Chl *a* Concentrations and Microbial Community Structure

Chlorophyll *a* (Chl *a*) samples were collected from 16 layers in the upper 200-m layer, including 10 fixed depths, 5 light depths, and a subsurface chlorophyll maximum (SCM) layer. The SCM layer was determined from *in situ* Chl *a* fluorescence measurements using a fluorometer equipped to the CTD system. Samples were filtered on 25-mm GF/F filters, and Chl *a* concentrations were determined fluorometrically (Welschmeyer, 1994) using a fluorometer (10AU Field and Laboratory Fluorometer; Turner Design, San Jose, CA, United States) after extraction in N, N-dimethylformamide (Suzuki and Ishimaru, 1990).

Microbial community structures were determined at the same 5 light depths as the Chl *a* measurements. Samples were measured on-board immediately after sampling without cell fixation using a flow cytometer (CyFlow Space; Sysmex Partec, Germany). SYBR Green I was applied for staining bacterial cells, and autofluorescent cells were grouped into *Prochlorococcus*, *Synechococcus*, nanoeukaryotes, and picoeukaryotes.

### Dissolved Nutrients and Data Collection

We analyzed the nanomolar level nitrate + nitrite (N+N) and phosphate at Stations 4–17 using a highly sensitive colorimetric method (Hashihama et al., 2009). Unfiltered seawater samples were collected in 30 mL polypropylene tubes (Sarstedt, Nümbrecht, Germany) and analyzed on-board immediately after sampling. The detection limit was 3 nM for both N+N and phosphate. Arsenic interference on phosphate determination was negligible because it was below the detection limit (Hashihama et al., 2013). For micromolar level analyses, samples were collected from all stations in 10-mL acrylic spitz tubes and kept at −20°C until analysis on land using an AACS-II autoanalyzer (SPX FLOW Technology, Norderstedt, Germany). Micromolar level data sets were obtained at Stations 2 and 3 and for samples exceeding 1 μM by the sensitive method. Due to the lack of nutrient data sets for several depths at Station 3, we assumed their concentration based on the interpolation of the vertical depth profiles.

As for the determination of labile monoesters and diesters, we applied an enzymatic hydrolysis method (Suzumura et al., 1998; Hashihama et al., 2013; Yamaguchi et al., 2019)

on 30 mL of unfiltered seawater samples. In brief, two types of working enzyme solutions were prepared containing monoesterase (P-4252; Sigma-Aldrich) or [mono- + diesterase (Phosphodiesterase I; Worthington)] at final concentrations of 0.2 U mL^–1^. Samples were added with either of the working enzyme solutions, 0.5 M Tris buffer solution, and 5 M sodium azide solution. Samples were then frozen to terminate enzyme activity after 5 h of dark incubation at 30°C. Nanomolar monoester and diester concentrations were measured as phosphate concentrations increased from blank samples (Hashihama et al., 2009). Blank samples were prepared in a similar manner but without the addition of working enzyme solution and Tris buffer. Finally, the results were corrected for sample dilution by the added reagents and phosphate contamination therein. Phosphate contamination was determined using MAGIC seawater (Karl and Tien, 1992) as a phosphate-free matrix. The detection limit for monoester and diester concentrations was 3.4 nM (Yamaguchi et al., 2019), and the precision was ±1.7 nM (Hashihama et al., 2013). The hydrolysis efficiency of this determination method was previously evaluated using several known phosphoric esters (Hashihama et al., 2013; Yamaguchi et al., 2019).

Total dissolved phosphorus (TDP) concentrations were measured by the persulfate oxidization decomposition method (Hansen and Koroleff, 1999). TDP samples were filtered through precombusted GF/F filters (450°C, 3 h) into polypropylene tubes and kept frozen at −20°C until analyses on land. Decomposed TDP concentrations were measured as phosphate at the nanomolar level when the background phosphate concentrations were below 500 nM. Therefore, the data sets were partly lacking at stations with high ambient phosphate concentrations. DOP concentrations were defined as TDP minus phosphate.

All nutrient samples were generally collected at the same 16 depths as the Chl *a* samples at all stations. Nutrient concentrations below the detection limit were considered 0 nM when calculating their means and integrals, and when producing plots via Ocean Data View. For convenience, the zero values were substituted by the detection limit when deriving the non-linear power function regressions.

The data and methodologies from 12 references on enzymatically hydrolyzed labile phosphoric esters in various aquatic environments are summarized in Table 1. In the absence of precise information on raw values, they were estimated from the provided graphs and bar charts using WebPlotDigitizer Version 4.2 (Ankit Rohatgi, San Francisco, CA, United States).

**TABLE 1:** Summary data of labile phosphoric esters.

Sampling area	Sampling period	Vertical data		Horizontal data		Detection limit	Assayed fraction	Surface phosphate	Azide addition	Monoester			Diester			References
										Enzyme origin	Concentration	%DOP	Enzyme origin	Concentration	%DOP	
**North Pacific**												
western region	July	Yes	0–200 m	Yes	137°E 30–33°N	3 nM	0.45 μm filtrate	10–23 nM	Yes	bovine intestine	18–55 nM	14–48%	nd	nd	nd	Suzumura et al. (2012)
western region	July	Yes	0–200 m	Yes	137°E 30–33°N	3 nM	unfiltered SW	11–22 nM	Yes	*Escherichia coli*	< 62 nM	< 27%	nd	nd	nd	Hashihama et al. (2013)
western region	July, August	discrete	10 m, SCM	Yes	133–160°E 10–38°N	3 nM	unfiltered SW	< 89 nM	Yes	*E. coli*	3–21 nM	< 20%	nd	nd	nd	Sato et al. (2013); Hashihama et al. (2019)
central region	July	No	15–20 m	Yes	139–147°W 30–32°N	5 nM	unfiltered SW	13–59 nM	No	*E. coli*	< 5 nM	nd	nd	nd	nd	Duhamel et al. (2010)
central region	June–August	Yes	0–170 m	Yes	170°W 5–40°N	3.4 nM	unfiltered SW	20–163 nM	Yes	*E. coli*	< 40 nM	< 40%	*Crotalus adamanteus* venom	< 40 nM	< 100%	Yamaguchi et al. (2019)
central region	December, January	discrete	< 10 m, SCM	Yes	155°E–158°W 23°N	3 nM	unfiltered SW	< 51 nM	Yes	*E. coli*	3–25 nM	< 15%	nd	nd	nd	Sato et al. (2013); Hashihama et al. (2019)
subtropical region	August–October	Yes	0–170 m	Yes	137°E–120°W 23–30°N	3.4 nM	unfiltered SW	< 280 nM	Yes	*E. coli*	< 41 nM	< 65%	*C. adamanteus* *venom*	< 21 nM	< 47%	This study
**South Pacific**																
eastern region	November, December	No	5 m	Yes	90–130°W 10–35°S	20 nM	unfiltered SW	127 nM	No	*E. coli*	< 20 nM	nd	nd	nd	nd	Moutin et al. (2008)
subtropical region	December, January	discrete	< 10 m, SCM	Yes	100–170°W 17–35°S	3 nM	unfiltered SW	21–194 nM	Yes	*E. coli*	3–86 nM	< 72%	nd	nd	nd	Sato et al. (2013); Hashihama et al. (2019)
**North Atlantic**																
central region	March, April	No	35–100 m (SCM only)	Yes	30–54°W 29–40°N	10 nM	HMW DOP (> 1 kDa)	< 120 nM	No	bovine intestine	11–21 nM	10–30%	*C. adamanteus* *venom*	4–11 nM	4–16%	Vidal et al. (2018)
**Non-pelagic regions**																
Tokyo Bay	July–October	No	0 m	Yes	139–140°E 35–36°N	∼10 nM	HMW DOP (> 10 kDa)	0.25–6 μM	Yes	bovine intestine	6–24 nM	< 14%	*C. adamanteus* *venom*	17–59 nM	< 19%	Suzumura et al. (1998)
Sagami Bay	July	Yes	0–50 m	No	139.3°E 35°N	20 nM	0.45 μm filtrate	< 20 nM	Yes	bovine intestine	24–52 nM	32–85%	nd	nd	nd	Suzumura et al. (2012)
continential shelf	July	Yes	0–50 m	No	137°E 34°N	3 nM	unfiltered SW	5 nM	Yes	*E. coli*	6–243 nM	nd	nd	nd	nd	Hashihama et al. (2013)
East China Sea	September	Yes	0–70 m	Yes	127–130°E 28–31°N	3 nM	unfiltered SW	22–25 nM	Yes	*E. coli*	14–75 nM	nd	nd	nd	nd	Hashihama et al. (2013)
Tamar estuary	All seasons	No	0.3 m	Yes	4–5°W 50–51°N	0.2 μg P/L (~6.5 nM)	0.2 μm filtrate	8.8–61 μg P/L (0.28–2.0 μM)	No	calf intestine	< 9.7 μg P/L (~313 nM)	< 79%	*C. atrox* *venom*	< 7.3 μg P/L (~236 nM)	< 100%	Monbet et al. (2009)
Florida Bay	All seasons	No	< 2 m	Yes	80–82°W 24–26°N	20 nM	0.2 μm filtrate	36–365 nM	No	calf intestine	< 64 nM	< 100%	nd	nd	nd	Koch et al. (2009)
Toulon Bay	All seasons	No	3 m	Yes	5–6°E 43–44°N	nd	0.45 μm filtrate	< 185 nM	No	calf intestine	< 124 nM	< 100%	nd	nd	nd	Bogé et al. (2014)

*Surface phosphate shows either phosphate concentrations at 0 m or the shallowest depth. %DOP shows the ratio of each ester against total dissolved organic phosphorus (DOP) (nd, no data; SW, sea water; HMW, high molecular weight).*

### Kinetic Parameters of Alkaline Phosphatase Activity

Samples for alkaline phosphatase activities were collected from the same 5 light depths at all stations. Potential *V*_max_, *K*_m_, and turnover times (*T*_n_) of bulk MEA and DEA were determined by multiple-concentration assays (Sato et al., 2013). Two types of artificial fluorescent substrates were applied – 4-methylumbelliferyl phosphate (MUP; Thermo Fisher) and bis-MUP (Chem-Impex Int. Inc.) – and 50 μM of each stock solution was prepared separately in autoclaved 3.5% NaCl aqueous solution. The stock solutions were then kept frozen until later use. Either of these stock solutions was added separately to 2.5 mL of unfiltered seawater to final concentrations of 0.1, 0.5, 1, or 2 μM for the MEA assay and 0.1, 0.5, 1.5, 3, or 4 μM for the DEA assay. Blank samples were prepared in the same manner without the addition of fluorescent substrates.

The fluorescence intensities of hydrolyzed products (4-methylumbelliferone, MUF) were measured using a spectrofluorometer (FP-8200; JASCO, Tokyo, Japan). Measurements were taken immediately after substrate addition and several times during the 9–11 h of dark incubation at *in situ* temperatures to ensure linear hydrolysis rates. MUF standard solutions were prepared at each sampling station using GF/F filtered surface seawater as a solvent. The standard solutions were incubated in the same manner as the other samples, and the MUF standard curves were obtained at each measuring time point.

Based on the time-course hydrolysis rates, we calculated the *V*_max_ and *K*_m_ for MEA and DEA by curve fitting the Michaelis–Menten equation using the “Ligand binding” package in SigmaPlot 9.0 (Systat Software Inc., San Jose, CA, United States) (*p* < 0.05). However, the derived *V*_max_ and *K*_m_ for DEA were partly insignificant due to poor regression. Therefore, we estimated the missing DEA *V*_max_ data from the relevant hydrolysis rates at the highest bis-MUP concentration as they showed a linear relationship with the corresponding DEA *V*_max_ data (*p* < 0.001, data not shown) within the data set deriving significant regression curves. The potential *T*_n_ of MUP and bis-MUP, identified as those of *in situ* monoesters and diesters, were derived by dividing *K*_m_ by *V*_max_ (Labry et al., 2005).

Three size fractions of MEA and DEA were determined at all stations except for Station 17: (1) the dissolved fraction (<0.2 μm; MEA_dis_, DEA_dis_), (2) the small particulate associated fraction (0.2–0.8 μm; MEA_small_, DEA_small_), and (3) the large particulate associated fraction (>0.8 μm; MEA_large_, DEA_large_). Seawater samples were filtered through DISMIC filters (ADVANTEC, Japan), and the same fluorescent substrates were added to final concentrations of 2 and 4 μM for the MEA and DEA assay, respectively. Incubation and measurement procedures were done similarly to those above and the proportion of each fraction to the bulk hydrolysis rate was calculated. Note that the results of the size-fractionated DEA at Station 11 were excluded as outliers because they were unaccountably higher than the bulk DEA.

## Results

### Environmental Descriptions and Regional Divisions

**FIGURE 2:**
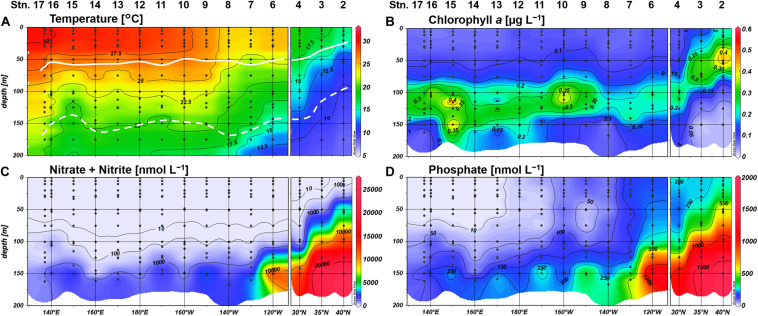
Distributions of **(A)** temperature, **(B)** chlorophyll *a* concentrations, **(C)** nitrate+nitrite concentrations, and **(D)** phosphate concentrations. Light depths of 10% and 0.1% are indicated by white bold and dashed lines, respectively.

The northeastern three stations off the California coast (Stations 2–4) were clearly distinguishable from other stations, showing typically low temperature (Figure 2A). Moreover, shoaling of nutriclines was notable at Stations 2 and 3, resulting in a shallow SCM above 70-m depth (Figures 2B–D). We observed a westward increase in temperature along the 23°N transect (Figure 2A), and the water column stratification was similar for all stations west of 160°W. N+N concentrations were mostly depleted (<5 nM) in the upper 100-m layer, and the concentrations around the bottom of the euphotic zone decreased westward (Figure 2C). A similar trend in the deeper layers was also observed for phosphate concentrations (Figure 2D). However, phosphate concentrations showed a distinct east-west gradient, with severe depletion (<3 nM) in the shallow layer (defined hereafter as ≤75 m) at Stations 13–17. The SCM located adjacent to the nitracline at 100 to 135-m depth (Figure 2B).

**FIGURE 3:**
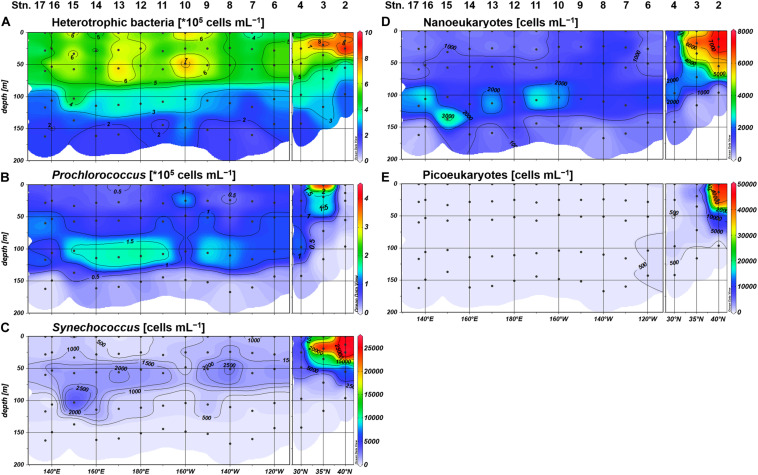
Distributions of the cell abundances of **(A)** heterotrophic bacteria, **(B)** *Prochlorococcus*, **(C)** *Synechococcus*, **(D)** nanoeukaryotes, and **(E)** picoeukaryotes.

We observed similar geographical contrasts in microbial community structure between the northeastern and the subtropical regions. *Synechococcus*, nanoeukaryotes, and picoeukaryotes were substantially higher in abundance among the phytoplankton community at Stations 2 and 3, whereas the proportion of *Prochlorococcus* abundance was higher in the subtropical region including Station 4 (Figure 3). Heterotrophic bacteria, *Synechococcus*, and picoeukaryotes were frequently observed in the upper euphotic zone (defined hereafter as ≥10% light intensity), while *Prochlorococcus* and nanoeukaryotes generally peaked at 1% light depth in the subtropical region (Figure 3). Based on the distributions of inorganic nutrients, SCM depths, and microbial community compositions, we divided the study area into the northeastern (Stations 2 and 3) and subtropical (Stations 4–17) regions.

### Distributions of DOP Compounds

**FIGURE 4:**
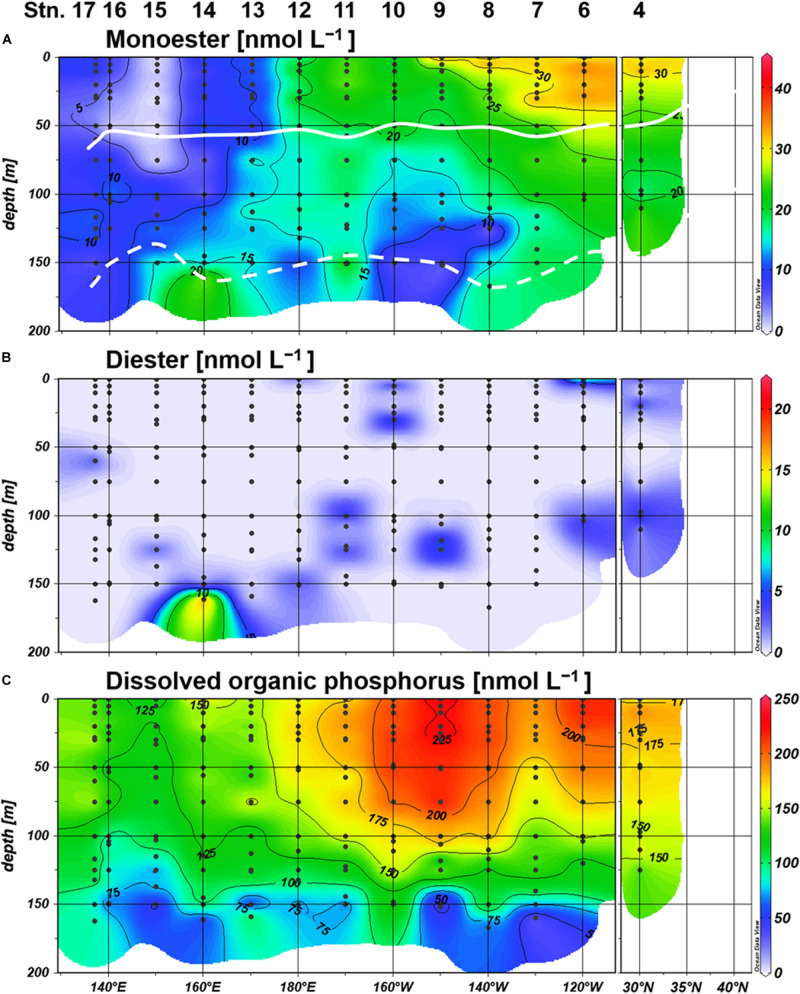
Distributions of **(A)** monoester, **(B)** diester, and **(C)** dissolved organic phosphorus (DOP) concentrations. Light depths of 10% and 0.1% are indicated by white bold and dashed lines, respectively.

**FIGURE 5:**
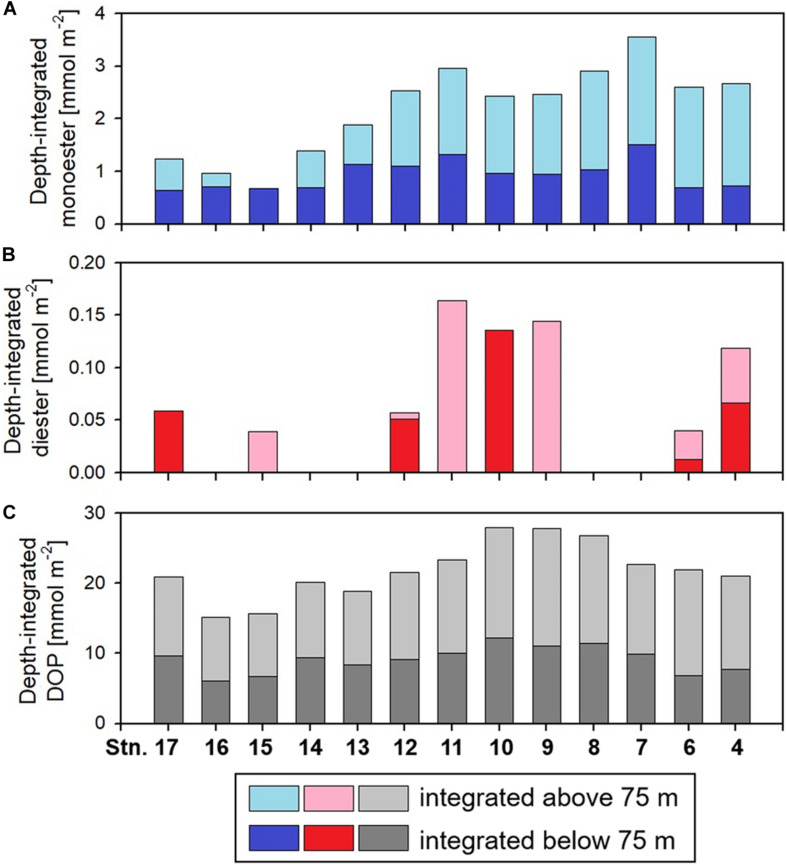
Distributions of the depth-integrated amount of **(A)** monoester, **(B)** diester, and **(C)** dissolved organic phosphorus (DOP). At the two eastern stations 4 and 6, these parameters were integrated only above 110 and 104 m, respectively, due to lacking data sets.

**FIGURE 6:**
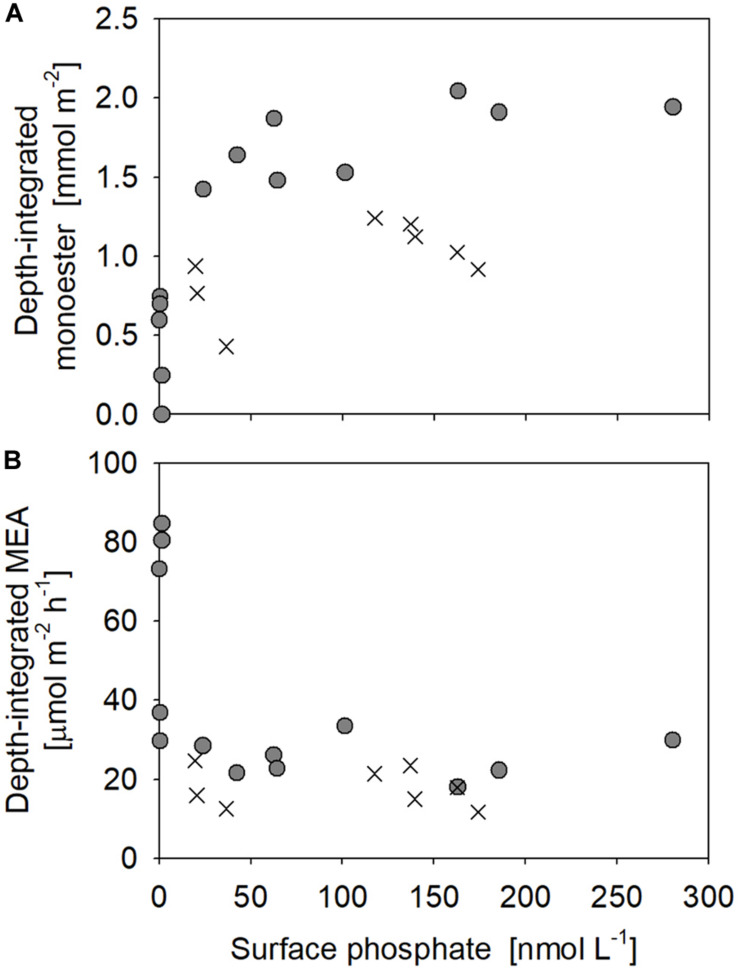
Relationships between surface phosphate concentrations vs. depth-integrated **(A)** monoester concentrations and **(B)** monoesterase activity (MEA) above 75 m. Filled circles represent the results obtained in this study (Stations 4–17), whereas crosses are those from Yamaguchi et al. (2019).

Labile monoester concentrations ranged from undetectable levels to 41 nM, averaging 16 ± 9 nM (*n* = 198) (Figure 4A). The depth-integrated monoesters above 150 m gradually decreased westward, following the east-west gradient in phosphate concentrations. We observed a maximum fivefold difference between Stations 7 and 15 (Figure 5A). As we observed no longitudinal variation in the depth-integrated monoesters below 75 m (Smirnov-Grubbs test, *p* > 0.05), the east-west variability likely resulted from the fluctuation of monoester concentrations in the shallow layer, whose depth-integrated values showed an increase against surface phosphate concentrations (Figure 6A). Monoester concentrations were generally below 10 nM in the shallow layer of the phosphate-depleted region (Stations 13–17) and were particularly depleted at Station 15 (Figure 4A).

**FIGURE 7:**
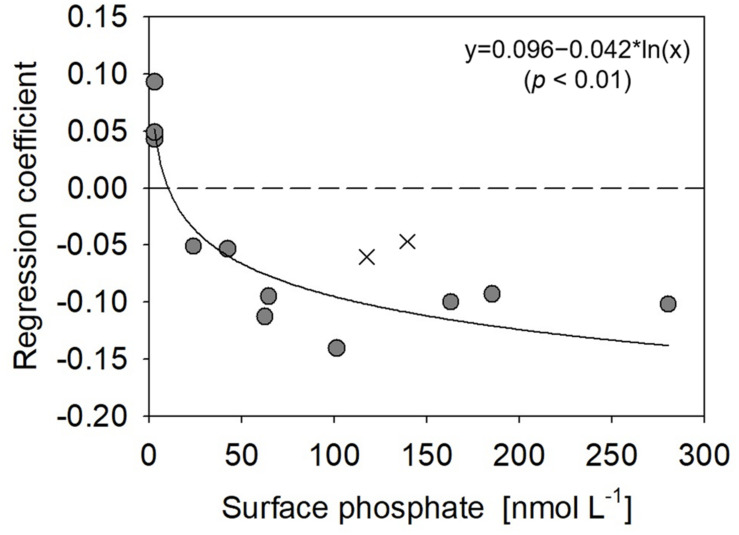
Relationship between surface phosphate concentrations vs. regression coefficients derived between monoester concentrations and depths at stations 4–15. Filled circles represent the results obtained in this study, whereas crosses are those from Yamaguchi et al. (2019).

The vertical profiles of monoester concentrations also demonstrated longitudinal variability. Monoester concentrations were high around the sea surface and decreased with depth in the eastern half of the transect (Stations 4–12) (Figure 4). They were significantly negatively correlated with depth at each station, and these vertical profiles were opposite to those of other inorganic nutrients (Figure 2). In contrast, significant positive correlations were observed at Stations 13–15. Interestingly, the regression coefficients were significantly negatively correlated with surface phosphate concentrations (Figure 7). From this non-linear regression, the threshold surface phosphate concentration separating the depth-negative and depth-positive correlation of monoesters was 10 nM. Monoester concentrations subtly increased with depth at Stations 16 and 17, although the correlation was not significant.

Labile diester concentrations ranged from undetectable levels to 21 nM (*n* = 200) (Figure 4B). Over 90% of samples were under the detection limit and the average value calculated from detectable results was 8 ± 4 nM (*n* = 17). Due to this scarcity, the depth-integrated diesters were an order of magnitude lower than that of monoesters (Figure 5B), and the diester concentrations showed no obvious geographical variation (Figure 4B).

Dissolved organic phosphorus concentrations showed an opposite depth profile to that of phosphate (Figure 4C), and the depth-integrated concentrations were particularly high at Stations 8–10 (Figure 5C). The DOP:TDP ratio gradually increased westward, reaching above 90% in the phosphate-depleted region (Stations 13–17) (Supplementary Figure S1A). The average contribution of monoester and diester concentrations to the total DOP was 11 ± 8% and 1 ± 4%, respectively. In the shallow layer, monoester:DOP ratios were lowest in the western region (Stations 13–17), followed by the mid-region (Station 8–12), and highest in the eastern region (Station 4–7) (Kruskal–Wallis test, *p* < 0.01; Supplementary Figure S1B).

### Distributions of Alkaline Phosphatase Activities

**FIGURE 8:**
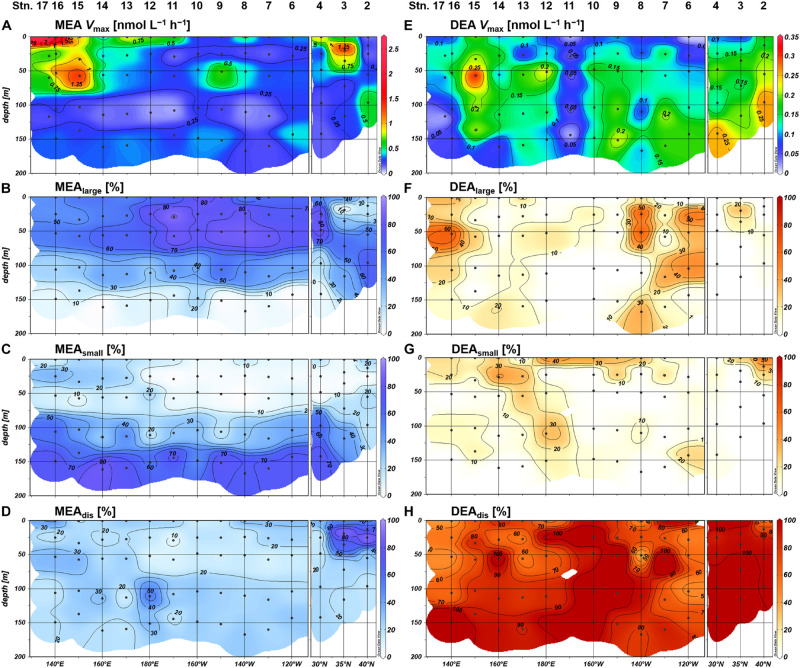
Distributions of monoesterase activity (MEA), diesterase activity (DEA), and the proportion of size-fractionated enzyme activities; **(A,E)** bulk enzyme activities in whole seawater samples, **(B,F)** enzyme activities in large size fraction (>0.8 μm), **(C,G)** enzyme activities in small size fraction (0.2–0.8 μm), and **(D,H)** enzyme activities in the dissolved fraction (<0.2 μm).

**FIGURE 9:**
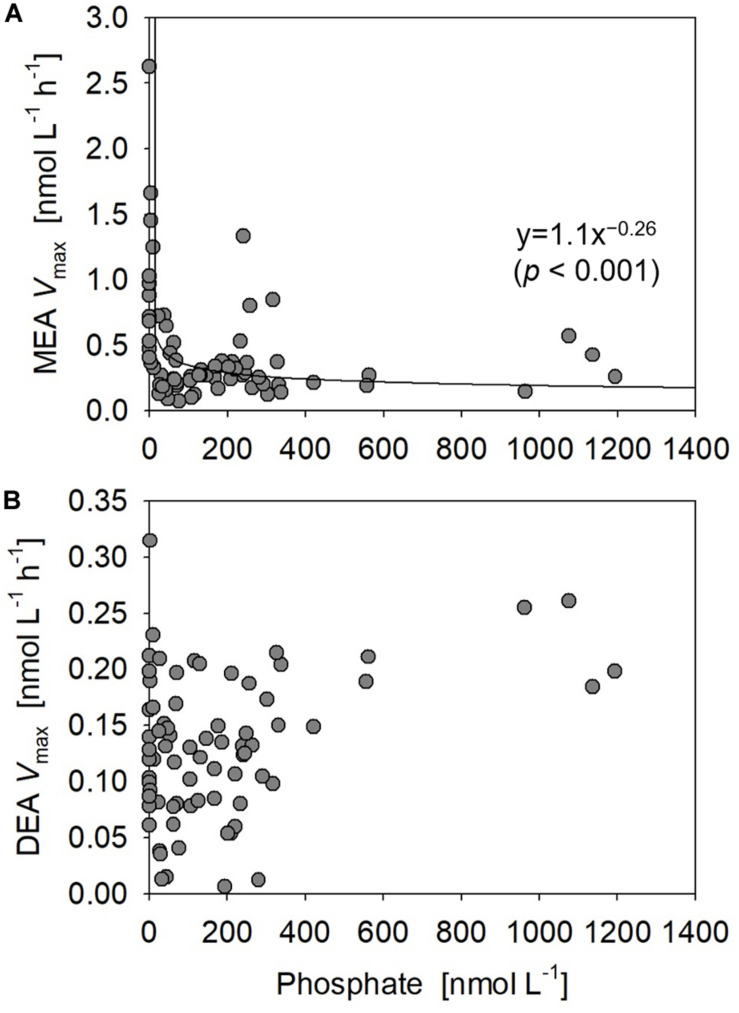
Relationships between phosphate concentrations vs. **(A)** monoesterase and **(B)** diesterase activities (MEA and DEA *V*_max_) obtained at all stations.

Monoesterase activity *V*_max_ and *T*_n_ varied between 0.075 and 2.6 nmol L^–1^ h^–1^ (mean 0.50 ± 0.43 nmol L^–1^ h^–1^, *n* = 89) and 4–431 days (mean 64 ± 61 days, *n* = 89), respectively. MEA was generally upregulated in the upper euphotic zone of the study area (Figure 8A) and was significantly negatively correlated with phosphate concentrations (*p* < 0.001) (Figure 9A). The enhancement of MEA under low phosphate conditions was also notable from the depth-integrated MEA in the shallow layer (Figure 6B). MEA was particularly high exceeding 1 nmol L^–1^ h^–1^ at Stations 15–17 (Figure 8A), with a notably short *T*_n_ below 10 days. MEA was also high at Station 3, although *T*_n_ was substantially longer (>85 days) than in the phosphate-depleted region (Stations 13–17). In addition, MEA was significantly negatively correlated with monoester concentrations (Supplementary Figure S2); however, two data sets were identified as outliers due to their large residual errors exceeding 2σ.

The size fractionation of enzyme assays revealed that MEA_large_ accounted for the largest proportion of the bulk MEA on average (48 ± 26%; *n* = 70), whereas MEA_small_ and MEA_dis_ showed similar average contributions of 26 ± 24% and 26 ± 16%, respectively. The contributions of MEA_large_ and MEA_small_ showed mostly inverse vertical trends throughout the study area and dominated the bulk MEA in the upper and deeper (≤1% light intensity) euphotic zones, respectively (Figures 8B,C). Exceptions to these trends were observed in the northeastern region (Stations 2 and 3) where MEA_dis_ showed the highest contribution in the upper euphotic zone relative to MEA_large_ (Figure 8D). From a horizontal viewpoint, the MEA_small_ contribution increased whereas the MEA_large_ contribution decreased in the upper euphotic zone of the phosphate-depleted region (Stations 13–17) (Figures 8B,C). The MEA_dis_ contribution was consistently low throughout the subtropical region; however, we observed a slight increase in the upper euphotic zone of the phosphate-depleted region (Figure 8D).

Diesterase activity *V*_max_ and *T*_n_ varied between 0.006 and 0.31 nmol L^–1^ h^–1^ (mean 0.13 ± 0.064 nmol L^–1^ h^–1^, *n* = 89) and 956–1529 days (mean 1268 ± 210 days, *n* = 15), respectively. Note that DEA *T*_n_ was seldom determined due to the poor fitting of regression curves for uncertain reasons. Unlike the features of MEA, DEA was rarely upregulated at the sea surface and peaked around 10% light depth in the phosphate-depleted region (Stations 13–17) (Figure 8E). Furthermore, DEA increased with depth, particularly in the eastern half of the study area (Stations 2–10). Thus, we identified no significant relationship between the bulk DEA and phosphate concentrations (Figure 9B).

The characteristics of size-fractionated DEA were also distinct from those of MEA. Notably, DEA_dis_ was the dominant fraction of the bulk DEA accounting for 85 ± 24% (*n* = 64) on average, whereas DEA_large_ and DEA_small_ accounted for only 13 ± 17% and 12 ± 14% on average, respectively. Both the percentage and absolute values of DEA_large_ and DEA_small_ were significantly negatively correlated with phosphate concentrations (Supplementary Figure S3), except for the excluded high-value DEA_small_ outlier at Station 2. However, DEA_large_ and DEA_small_ showed different distributions in the subtropical region: DEA_large_ was high at Stations 6–8 and 15–16, whereas DEA_small_ was notable at Stations 9–14.

## Discussion

### Quantification of Bioavailable DOP

To date, several methodologies, including enzymatic degradation, photodegradation, and estimation from radiotracer uptake, have been developed to quantify the bioavailable DOP pool considering its importance to the physiological ecology and biogeochemical cycles. However, there is still a fundamental caveat in these methodologies as the components of the determined fractions are mostly unknown. This leads to a query of whether they are actually utilized *in situ* and to what extent they account for the genuine bioavailable DOP. Moreover, the ratios of quantified amounts of labile DOP against total DOP largely depend on their methodological definitions. For example, these ratios tend to be higher when determined by photodegradation (average: 67%, Karl and Yanagi, 1997) and radiotracer experiments (average: 24%, Björkman et al., 2000) than by enzymatic degradation (average: 12%, this study) in the North Pacific. Moreover, even though enzymatic degradation is intended for phosphoric ester detection, the substrate specificity of enzymes is inevitably involved (Turner et al., 2002; Hashihama et al., 2013); therefore, our results should be regarded as the “minimum” values of the labile phosphoric ester pool. Thus, the unidentified DOP fraction in this study cannot be recognized as completely refractory as it likely involves both refractory and undetectable labile phosphoric esters, as well as bioavailable polyphosphates and phosphonates (Björkman and Karl, 1994; Dyhrman et al., 2006).

### Dynamics of Monoesters

#### Regulation of Monoester Distribution

Our basin-scale investigation is the first to report the geographical variations of phosphoric monoester and diester concentrations along a vast longitudinal transect in the subtropical North Pacific. Despite the narrow range and low values at nanomolar levels of monoester concentrations in our study area, we observed an obvious geographical gradient and identified novel features in the phosphate-replete eastern subtropical North Pacific. The distribution patterns were most likely regulated by ambient phosphate concentrations. Severe phosphate depletion (under ca. 10 nM) in the shallow layer of the western basin led to an extremely low level of monoesters, resulting in a depth-increasing profile. In contrast, higher phosphate concentrations in the shallow layer of the eastern basin led to higher monoester concentrations, resulting in a depth-decreasing profile.

Within our study area, Stations 13–17 were severely P-limited due to the exhaustion of phosphate in the shallow layer. This phosphate depletion was accompanied by severely low monoester concentrations below 10 nM. Simultaneously, high MEA in the upper euphotic zone (Figures 8A, 9A) clearly demonstrated the active utilization and exhaustion of monoesters as alternative microbial P sources; this most likely induced the depth-increasing trends of monoesters at Stations 13–15. The depth-increasing trend was relatively obscure in the westernmost region (Stations 16 and 17); however, we assume that these depth profiles were a derivative of the neighboring distribution patterns, as the monoester concentrations in the shallow layer were similarly low (Figure 4A).

In the eastern basin (Stations 4–12), monoester concentrations in the shallow layer increased in line with the eastward increase in ambient phosphate concentrations (Figures 2D, 4A). However, the increase in monoester concentrations cannot be simply explained by the fluctuation of total DOP alone, as we identified differences in the spatial variability of maximum DOP concentrations and monoester:DOP ratios (Figure 5C and Supplementary Figure S1B). Thus, labile monoesters were likely regulated by a different mechanism to that of the remaining fraction of total DOP. Because the standing stock of monoesters results from the equilibrium of both consumption and production processes, it is difficult to fully understand the dynamics due to lack of either data. However, when considering the significant depth-decreasing trend in monoester concentrations at all eastern stations, the relatively low MEA in the upper euphotic zone therein (Figure 8A) may have in part contributed to the possible imbalance and subsequent net accumulation of monoesters in the shallow layer.

#### Comparisons of Monoester Distributions Between Various Oceanic Regions

As summarized in Table 1, there have been several studies providing both vertical and horizontal distribution data of monoesters in the North Pacific (Table 1). While previous studies were limited to the central or western North Pacific, our study provides the data for the phosphate-replete eastern subtropical region. Moreover, our study covers a wider range of surface phosphate concentrations compared to previous studies, varying from undetectable levels to 280 nM (Table 1). Despite the variable range in ambient phosphate concentrations, monoester concentrations fell within the range of previous studies (Table 1); this suggests that monoester concentrations are consistently low at the nanomolar level in the subtropical ocean. In contrast, monoester concentrations are occasionally higher than 100 nM in non-pelagic regions (Table 1).

The characteristics of monoester distribution in the severely phosphate-depleted region in this study (i.e., low concentrations in shallow layers and the depth-increasing trends) were mostly consistent with those of previous studies conducted in the western North Pacific (Suzumura et al., 2012; Hashihama et al., 2013). This suggests that monoester distribution patterns are generally ubiquitous in this region. However, monoester concentrations were not completely exhausted in the findings reported by Suzumura et al. (2012) (Table 1), and their results differed to those of Hashihama et al. (2013), despite being conducted at the same sites and on the same cruise. These differences are likely due to discrepancies in their methodologies – such as applying sample filtration and monoesterase of different origins (Table 1). The origins of phosphatases are particularly essential because the pH optima and substrate specificities are known to differ among enzymes (Bünemann, 2008).

Under phosphate-replete conditions, we found an increase in the residual monoester concentrations with increasing ambient phosphate concentrations. This trend was consistent with the surface monoester concentrations reported along a longitudinal transect conducted in the winter (Sato et al., 2013; Hashihama et al., 2019; Table 1). Similar parameters were collected in these studies, and their sampling locations partly overlapped with ours which corresponded to Stations 10–14. From the paired comparisons, there was no obvious seasonality in monoester concentrations, as well as in phosphate and total DOP. MEA was significantly higher in our summer study (Wilcoxon signed-rank test, *p* < 0.05), although the fluctuation was within a moderate range. Therefore, the impact of MEA seasonality on the standing stock of P compounds was assumed to be small in the central North Pacific. However, because the comparable data sets were limited to particular seasons and depths, intensive seasonal investigations are recommended for further verification.

On the other hand, the coupled increase between phosphate and monoesters was not fully applicable to other previous studies in the central North Pacific and eastern South Pacific (Moutin et al., 2008; Duhamel et al., 2010; Yamaguchi et al., 2019). This was likely because the reported monoester concentrations in these studies were occasionally lower than expected when referring to our data at similar levels of surface phosphate concentrations (Table 1 and Figure 4A). Furthermore, Vidal et al. (2018) reported increasing monoester concentrations with decreasing phosphate concentrations in the North Atlantic, which is in contrast with the longitudinal trend observed in this study (Figure 4A).

However, the findings of each study cannot be adequately compared due to significant differences in the methodologies (Table 1). Firstly, for those lacking the addition of azide solution (Moutin et al., 2008; Duhamel et al., 2010), monoester concentrations are likely underestimated by bacterial uptake of released phosphate during sample incubation. This is because azide solution prevents bacterial activity (Feuillade and Dorioz, 1992) without interfering with phosphatase activities (Suzumura et al., 1998). Secondly, the sampling conditions by Vidal et al. (2018) varied significantly from those of ours, particularly with regards to limited sampling depths, the assayed fraction, and the use of monoesterases of different origins. Nevertheless, these studies provide a significant perspective on monoester dynamics, and further investigations and data collection are therefore necessary to minimize the existing methodological biases.

In contrast, our methodology was basically identical to that of Yamaguchi et al. (2019), as well as the aforementioned winter studies (Sato et al., 2013; Hashihama et al., 2019; Table 1). The horizontal and vertical characteristics of monoester distributions in both studies were partly consistent, with increasing depth-integrated monoester concentrations under phosphate-rich conditions (Figure 6A; Yamaguchi et al., 2019). Here monoesters were integrated above 75 m when considering the longitudinal variability revealed in this study (Figure 5A). From a vertical viewpoint, Yamaguchi et al. (2019) also reported higher monoester concentrations in the upper 100-m layer, and their regression coefficients against depth were plotted close to the curve obtained in this study (Figure 7). However, only 2 out of 8 stations showed significant depth regressions (Yamaguchi et al., 2019) although the surface phosphate concentrations were thoroughly above the threshold (Table 1). In addition, the depth-integrated monoesters were relatively lower than those observed in this study, despite the similar range of surface phosphate concentrations (Figure 6A). This suggests that the residual monoester concentrations in the shallow layer was not as large as expected in Yamaguchi et al. (2019). As the depth-integrated MEA above 75 m were generally similar between the two studies (Figure 6B), the obscure depth-decreasing trend of monoesters in the central North Pacific was probably caused by low monoester net production and not by their increased hydrolysis in the shallow layer.

#### Distribution of Size-Fractionated MEA

Based on the size fractionation of phosphatase activities, the major monoester consumers were present in the large size fraction (>0.8 μm) in the upper euphotic zone of the subtropical region (Stations 4–17). This size class most likely consists of *Synechococcus*, nanoeukaryotes, and others including microplankton groups and particle aggregates. The dominance of MEA_large_ in the subtropical region was rather unique because MEA_dis_ contributed over 65% of the bulk MEA in the central North Pacific (30–32°N; Duhamel et al., 2010). In contrast, MEA associated with the small size fraction (0.2–0.6 μm) contributed over 40% of the bulk MEA in the tropical North Pacific (10–17°N; Hoch and Bronk, 2007). Nevertheless, a latitudinal shift in the dominant MEA size class was reported in the Atlantic from 10° to 30° in both N and S, varying from MEA_small_ to MEA_large_ and then to MEA_dis_ (Vidal et al., 2003). Therefore, the high proportion of MEA_large_ in this study can be interpreted as a specific feature along the 23°N transect, although further latitudinal investigations are required.

Compared with MEA_large_, the proportion of MEA_small_ – which likely consists of *Prochlorococcus* and heterotrophic bacteria – relatively increased in the upper euphotic zone of the western basin. This may be due to an increased abundance and/or increased P demand of these small microbes relative to larger communities. In the upper euphotic zone, the abundance of heterotrophic bacteria was mostly higher than that of *Prochlorococcus* in both cell counts and estimated carbon biomass at factors of 6.3 and 35.4 fg C cell^–1^, respectively (Kawasaki et al., 2011). Moreover, cell-specific phosphate uptake rates of heterotrophic bacteria were reported to be lower than those of *Prochlorococcus* in the oligotrophic ocean (Michelou et al., 2011; Björkman et al., 2012), inferring a higher sensitivity of heterotrophic bacteria to P stress. Thus, the increased proportion of MEA_small_ in the upper euphotic zone indicates the preferential utilization of monoesters by heterotrophic bacteria under low phosphate conditions in the western basin.

As previously mentioned, Li et al. (1998) proposed a hypothetical model to estimate the history of microbial P limitation based on the balance of phosphatase activity between the dissolved and particulate fraction. According to their assumption, high MEA_dis_ with low particulate MEA indicates “recent relief” from P limitation on microbes (Li et al., 1998). This is because the lifetime of dissolved monoesterase is assumed to be longer than that of microbes, lasting for more than several days and weeks (Li et al., 1998; Baltar et al., 2013). Moreover, high MEA_dis_ with high particulate MEA indicates persisting P limitation, whereas low MEA_dis_ with high particulate MEA indicates recent P limitation (Li et al., 1998). In this sense, the relatively low contribution of MEA_dis_ throughout the subtropical region with a mean of 23% (Figure 8D) indicates some degree of microbial P-deficiency across the study area, including the phosphate-replete eastern basin, and the moderate increase of MEA_dis_ in the western basin is consistent with the severe P limitation implicated from other parameters.

Despite the large geographical gradient in ambient phosphate concentrations, MEA_dis_ seldom exceeded 50% of the bulk MEA in the subtropical region. We therefore could not explain the high variance of MEA_dis_ reported in previous studies (Vidal et al., 2003; Hoch and Bronk, 2007; Duhamel et al., 2010, 2011; Sato et al., 2013). In particular, Vidal et al. (2003) reported a high proportion of MEA_dis_ (40–100%) in the severely phosphate-depleted subtropical North Atlantic (Wu et al., 2000; Martiny et al., 2019). In this case, the dominance of MEA_dis_ cannot be interpreted as recent relief from P limitation. A possible explanation is the difference in the subcellular localization of enzymes in different gene families (Luo et al., 2009), with enhanced extracellular expression of PhoX phosphatase relative to PhoA and PhoD types. Moreover, a major diazotroph *Trichodesmium* was reported to enrich the transcription of PhoX in the Atlantic and PhoA in the Pacific (Rouco et al., 2018). It is therefore necessary to improve our understandings of the phosphatase gene families to clarify the variability of dissolved MEA in the subtropical ocean.

The northeastern region (Stations 2 and 3) likely have different mechanism for the regulation of MEA expression from that in the subtropical region. As the northeastern region was characterized by a distinct microbial composition and a shallow SCM, the high MEA_dis_ observed in the upper euphotic zone was likely liberated from these microbial communities. Moreover, the dominance of MEA_large_ in the deeper euphotic zone may be interpreted as MEA associated with sinking particles due to the high biomass in the upper layer. When considering the relatively high ambient phosphate concentrations and the long *T*_n_, the high bulk MEA likely resulted from the remineralization of dissolved and particulate organic matters for the acquisition of carbon rather than P. This assumption is supported by previous studies reporting high bulk MEA in nutrient-rich environments such as the deep sea (Koike and Nagata, 1997; Hoppe and Ullrich, 1999) and coastal areas (Taga and Kobori, 1978; Hoppe, 2003; Labry et al., 2016).

### Dynamics of Diesters

#### Distribution and Regional Comparisons of Diester Concentrations

A notable limitation currently exists in the diester methodology: the DEA assay targets semi-labile diester compounds, while the determination of diester concentrations targets a different group of *in situ* labile diester compounds (Yamaguchi et al., 2019). This discrepancy is inevitably caused by enzyme substrate specificity, as Phosphodiesterase I cannot hydrolyze the artificial fluorescent substrate bis-MUP applied in the DEA assay (Yamaguchi et al., 2019). In contrast, Phosphodiesterase I is known to fully hydrolyze DNA (Suzumura et al., 1998; Turner et al., 2002) and *p*-nitrophenyl thymidine 5′−monophosphate (Yamaguchi et al., 2019). As a result, the current common enzyme assay would underestimate *in situ* DEA, and the determined *in situ* labile diester concentrations are likely to involve nucleic-acid-like diester compounds.

Unlike monoesters, labile diester concentrations were mostly below the detection limit throughout the study area, despite the longitudinal gradient in ambient phosphate concentrations. The diester distribution was therefore assumed to be independent of phosphate, monoesters, and DOP. The observed scarcity of diesters in the study area is possibly due to either or both low production rates or high consumption. Although we lack the actual flux data, diesters were at least assumed to possess potentially high bioavailability (Yamaguchi et al., 2019), based on the shorter *T*_n_ of involved nucleic acids (<1 day, Brum, 2005) relative to that of monoesters herein (>4 days). Moreover, the *T*_n_ of phosphate in the North Pacific is likely to fall below 1 day when ambient phosphate concentrations are approximately below 10 nM (Björkman et al., 2018). Therefore, labile diesters are possibly the preferential P sources across the transect, regardless of ambient phosphate concentrations.

Our results are consistent with the findings of previous studies in other pelagic regions, with diesters fluctuating at extremely low levels under 40 nM (Table 1; Vidal et al., 2018; Yamaguchi et al., 2019). Diester concentrations are higher in non-pelagic regions, reaching above 230 nM (Table 1; Suzumura et al., 1998; Monbet et al., 2009), and this trend was similar to that of dissolved DNA (Karl and Bailiff, 1989 and references therein) as well as monoesters. Diester concentrations were generally lower than monoester concentrations (Table 1), except in Tokyo Bay (Suzumura et al., 1998), despite their large variability among different aquatic environments. It is therefore necessary to obtain further data to accurately determine the distinct and common features of labile diesters between regions and elucidate the underlying mechanisms.

#### Distribution of Size-Fractionated DEA

The bioavailability of semi-labile diesters indicated from the DEA assay was substantially lower than those of labile diesters and monoesters, as DEA was generally an order of magnitude lower than MEA. The estimated *T*_n_ of semi-labile diesters also exceeded 950 days, which was significantly longer than those of monoesters in this study and nucleic acids (Brum, 2005). Compared with the upregulation of MEA, DEA under low phosphate conditions was considered to be moderately upregulated from the absence of a clear relationship between the bulk DEA and phosphate (Figure 9B). These characteristics as well as the low bioavailability of semi-labile diesters in this study were consistent with previous studies in the North Pacific (Sato et al., 2013; Yamaguchi et al., 2019). However, the biomass-normalized DEA from these studies and the particulate-associated DEA in this study were both stimulated under phosphate-depleted conditions. These results demonstrate the importance of semi-labile diesters as alternative P sources in the subtropical ocean.

The distribution pattern of size-fractionated DEA highlights the differences in the regulation of diesterase and monoesterase. The high dominance of DEA_dis_ in this study suggests that they significantly contribute to the cycling of semi-labile diesters in the subtropical North Pacific. As the upregulation of particulate-associated DEA under phosphate depletion was masked by the fluctuation of DEA_dis_, the high proportion of DEA_dis_ can therefore be used as a proxy for past P stress – similar to that of MEA_dis_ (Li et al., 1998), although the lifetime of diesterase and their subcellular locations are not yet fully understood.

DEA_large_ and DEA_small_ were sensitive to the depletion of ambient phosphate concentrations (Supplementary Figure S3); however, their geographical variations were not coupled with those of MEA_large_ and MEA_small_, respectively (Figure 8). These results are confusing, as the subsequent hydrolysis of ester bonds by monoesterase to diesterase is essential for the complete removal of P from diesters. In addition, we did not observe a coupling between the distributions of DEA_large_ and DEA_small_, indicating that the utilization of semi-labile diesters benefited different microbial communities across the study area. For instance, the notable DEA_small_ at Station 2 coincided with a high abundance of picoeukaryotes (Figures 3E, 8G). Although we could not provide a robust explanation for these phenomena, the diesterase regulation appears to be more complex than monoesterase regulation, and we therefore recommend the application of size fractionation in DEA assay to verify on-going P stress in the pelagic oceans and to determine the microbial communities associated with diester utilization.

## Conclusion

This study revealed the geographical variability of various dissolved P compounds and alkaline phosphatase activities to assess microbial DOP utilization across a vast region covering the low latitudinal area in the North Pacific Ocean. Our basin-scale observation demonstrated a novel and fundamental characteristic of labile monoester distribution to show a longitudinal variability and depth-dependent profiles, which were most likely regulated by ambient phosphate concentrations. Monoesters were predominantly utilized by large microbes including phytoplankton, although they likely benefited heterotrophic bacteria particularly under phosphate-depleted conditions. Labile diesters were consistently scarce throughout the study area, and the cell-free DEA were significant in the remineralization of semi-labile diesters. While the regulatory mechanisms of phosphoric esters in our study may not be fully applicable to other local oceanic regions, we believe that our findings significantly contribute to the current understanding of marine P cycle and are beneficial for further laboratory and field investigations to elucidate the production processes of phosphoric esters.

## Data Availability Statement

The raw data supporting the conclusions of this article will be made available by the authors, without undue reservation.

## Author Contributions

TY, MS, FH, HK, TS, and HO designed and conducted the whole experiment. All authors discussed the results.

## Conflict of Interest

The authors declare that the research was conducted in the absence of any commercial or financial relationships that could be construed as a potential conflict of interest.
