# Supplementary material for: Longitudinal and Vertical Variations of Dissolved Labile Phosphoric Monoesters and Diesters in the Subtropical North Pacific
Source: Front Microbiol. 2021 Jan 20;11:570081. doi: 10.3389/fmicb.2020.570081 (PMC7854537; doi:10.3389/fmicb.2020.570081)
Supplement: Supplementary file 1 [file Data_Sheet_1.DOCX]

Supplementary Material

# Supplementary Figures

**
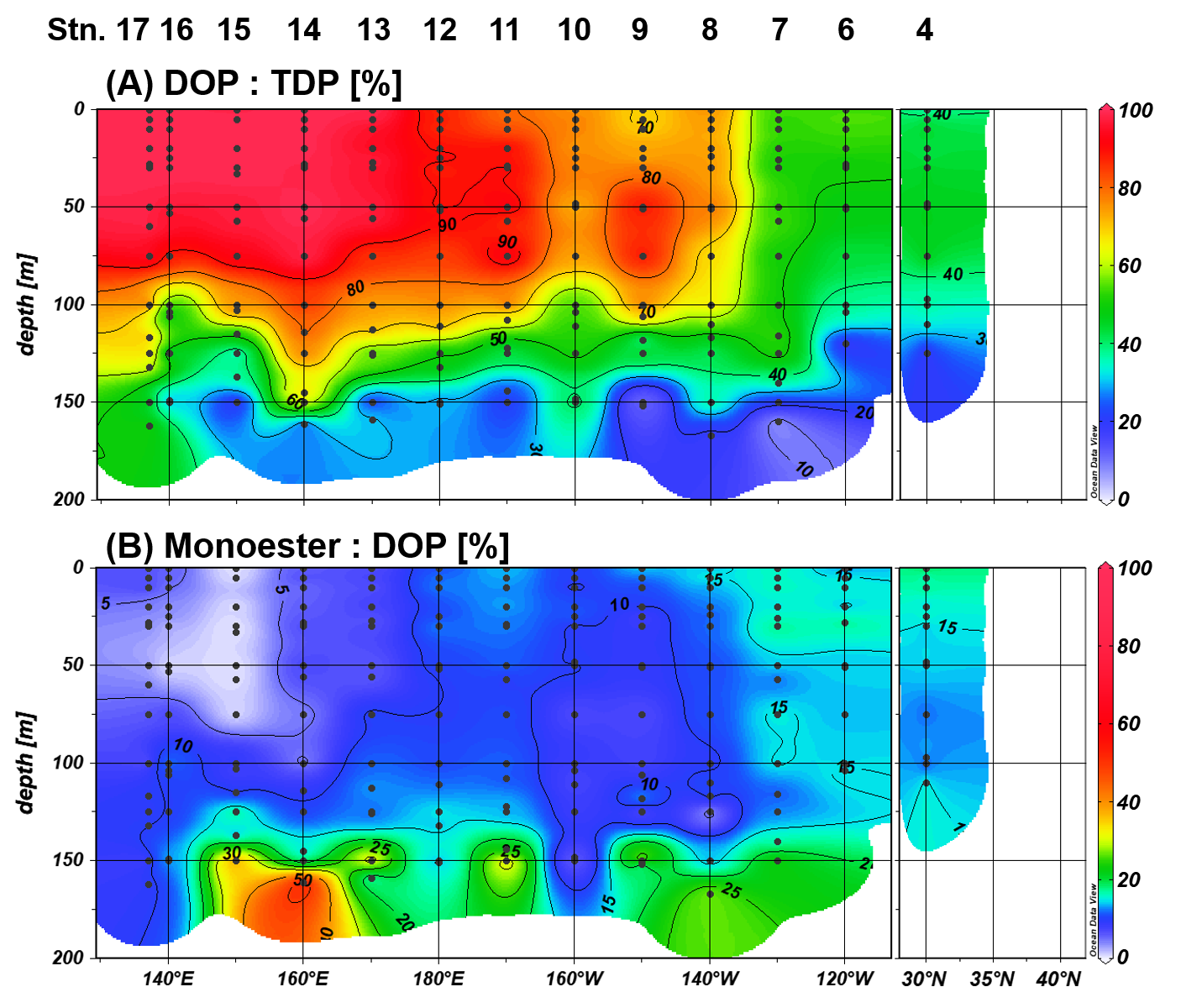
**

**Supplementary Figure 1.** Distributions of the proportions of (A) dissolved organic phosphorus (DOP) to total dissolved phosphorus (TDP) and (B) monoester to DOP.

**
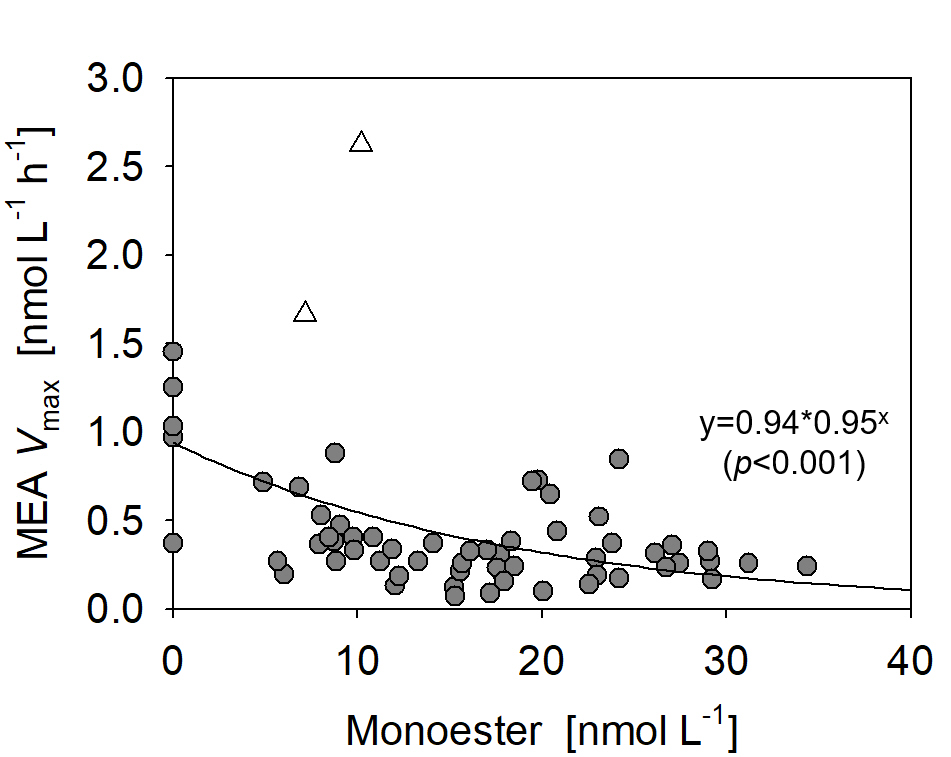
**

**Supplementary Figure 2.** Relationship between monoester concentrations vs monoesterase activity (MEA). Open triangles represent outlier data sets.

**
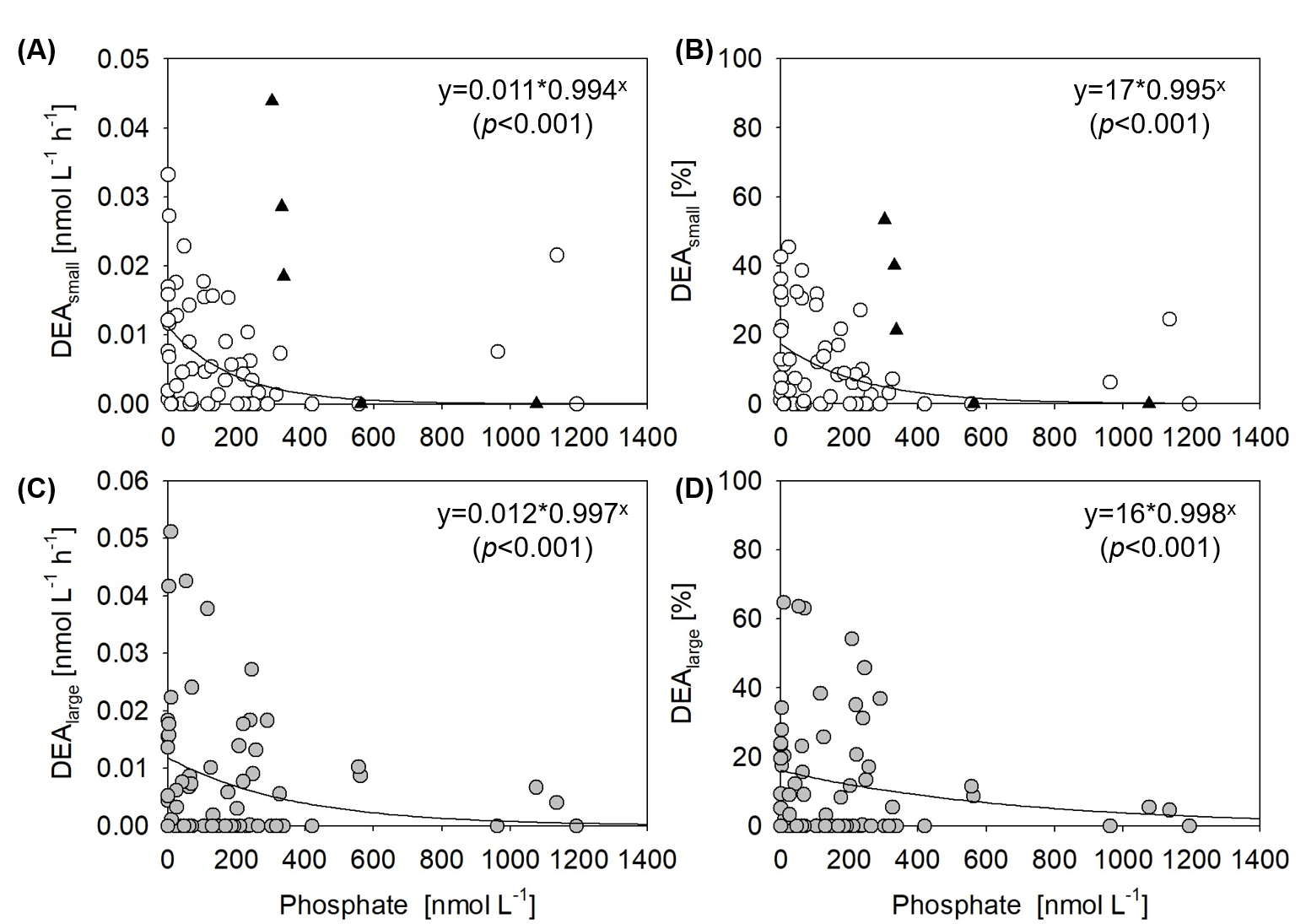
**

**Supplementary Figure 3.** Relationships between phosphate concentrations vs (A) diesterase activity in small size fraction (DEA_small_), (B) the proportion of DEA_small_ to bulk activity, (C) diesterase activity in large size fraction (DEA_large_), and (D) the proportion of DEA_large_ to bulk activity. Black triangles represent outliers for DEA_small_ obtained at station 2.
